# Ultrasound in cervical traumatic neuromas after neck dissection in thyroid carcinoma patients: descriptive analysis and diagnostic accuracy

**DOI:** 10.20945/2359-3997000000633

**Published:** 2023-05-29

**Authors:** Vinicius Neves Marcos, Debora Lucia Seguro Danilovic, Fernando Linhares Pereira, Miriam Harumi Tsunemi, Marco Aurelio Vamondes Kulcsar, Ana Oliveira Hoff, Regina Barros Domingues, Maria Cristina Chammas, Ricardo Miguel Costa de Freitas

**Affiliations:** 1 Universidade de São Paulo Instituto do Câncer do Estado de São Paulo Departamento de Radiologia São Paulo SP Brasil Universidade de São Paulo, Instituto do Câncer do Estado de São Paulo (Icesp), Departamento de Radiologia, Unidade de Ultrassom, São Paulo, SP, Brasil; 2 Universidade de São Paulo Instituto do Câncer do Estado de São Paulo Departamento de Endocrinologia São Paulo SP Brasil Universidade de São Paulo, Instituto do Câncer do Estado de São Paulo (Icesp), Departamento de Endocrinologia, São Paulo, SP, Brasil; 3 Universidade de São Paulo Instituto de Radiologia Departamento de Radiologia São Paulo SP Brasil Universidade de São Paulo, Instituto de Radiologia (InRad), Hospital das Clínicas, Departamento de Radiologia, Unidade de Ultrassom, São Paulo, SP, Brasil; 4 Universidade Estadual Paulista Departamento de Bioestatística Botucatu SP Brasil Universidade Estadual Paulista (Unesp), Departamento de Bioestatística, Botucatu, SP, Brasil; 5 Universidade de São Paulo Instituto do Câncer do Estado de São Paulo Departamento de Cirurgia de Cabeça e Pescoço São Paulo SP Brasil Universidade de São Paulo, Instituto do Câncer do Estado de São Paulo (Icesp), Departamento de Cirurgia de Cabeça e Pescoço, São Paulo, SP, Brasil; 6 Universidade de São Paulo Instituto do Câncer do Estado de São Paulo Departamento de Patologia São Paulo SP Brasil Universidade de São Paulo, Instituto do Câncer do Estado de São Paulo (Icesp), Departamento de Patologia, São Paulo, SP, Brasil

**Keywords:** Neuroma, ultrasonography, thyroid neoplasms, neck dissection

## Abstract

**Objective:**

Cervical traumatic neuromas (CTNs) may appear after lateral neck dissection for metastatic thyroid carcinoma. If they are misdiagnosed as metastatic lymph nodes (LNs) in follow-up neck ultrasound (US), unnecessary and uncomfortable fine-needle aspiration biopsy are indicated. The present study aimed to describe US features of CTNs and to assess the US performance in distinguishing CTNs from abnormal LNs.

**Subjects and methods:**

Retrospective evaluation of neck US images of 206 consecutive patients who had lateral neck dissection as a part of thyroid cancer treatment to assess CTN’s US features. Diagnostic accuracy study to evaluate US performance in distinguishing CTNs from abnormal LNs was performed.

**Results:**

Eight-six lateral neck nodules were selected for analysis: 38 CTNs and 48 abnormal LNs. CTNs with diagnostic cytology were predominantly hypoechogenic (100% *vs.* 45%; P = 0.008) and had shorter diameters than inconclusive cytology CTNs: short axis (0.39 cm *vs.* 0.50 cm; P = 0.03) and long axis (1.64 cm *vs.* 2.35 cm; P = 0.021). The US features with the best accuracy to distinguish CTNs from abnormal LNs were continuity with a nervous structure, hypoechogenic internal lines, short/long axis ratio ≤ 0.42, absent Doppler vascularization, fusiform morphology, and short axis ≤ 0.48 cm.

**Conclusion:**

US is a very useful method for assessing CTNs, with good performance in distinguishing CTNs from abnormal LNs.

## INTRODUCTION

Traumatic neuroma is a nonneoplastic nodular enlargement of a damaged nerve (1-3). Cervical traumatic neuromas (CTNs) may occur due to unintentional injury to the spinal nerves in 1.1% to 17.8% of lateral neck dissection for metastatic lymph nodes (LNs) (4-7). The CTNs present nodule-related pain between 11.1 and 40% patients (6,8) and surgical treatment is restricted to rare cases uncontrolled pain (9,10).

Postoperative cervical ultrasound (US) is recommended in the follow-up of patients with thyroid carcinomas (11,12). On US, CTN presents as heterogeneous nodular formation continuous to local cervical nerve, though it may not be easily recognized (6). Therefore, CTNs may be misdiagnosed as atypical LNs, leading to unnecessary and painful fine-needle aspiration biopsy (FNAB) (5-8). Additionally, cytology analysis confirms CTNs diagnosis in only 25 to 57.1% of cases (6,7), a fact that remains unexplained.

US evaluation of CTNs after lateral neck dissection is a rarely addressed topic (6-8). The aims of this study were to describe the B mode and color Doppler US features of CTNs and to assess the performance of US in distinguishing CTNs from abnormal cervical LNs in patients with thyroid carcinoma.

## MATERIALS AND METHODS

### Patient selection

From July 2016 to December 2017, 924 patients who underwent total thyroidectomy due to thyroid carcinoma were referred to the US unit of *BLINDED*. Consecutive cervical US exams from 206 patients who had complementary lateral neck dissection were selected and retrospectively reviewed. Patients whose US images were suggestive of CTNs or suspicious for lymph node metastasis were selected.

The US features considered suggestive of CTNs were either a nodule in continuity with a cervical spinal nerve or a fusiform nodule in posterolateral location in relation to the carotid artery without LN hilum characteristics (echogenic central line or central vascularization) (6-8). According to the aforementioned criteria, 79 patients (38.3%) were consecutively enrolled for US-guided FNAB. The diagnosis of CTN on FNAB consisted of the presence of neural spindle cells in cytology smear or, if non-diagnostic cytology, pain when inserting the needle into the nodule in addition to undetectable thyroglobulin or calcitonin in FNAB washout fluid (6,7). The visual analog scale of pain (VAS) (13) was applied for all patients before, immediately after, and 7 days after FNAB. The institutional Thyroid Cancer multidisciplinary team – including radiologists, endocrinologists and head and neck surgeons – decided to interrupt FNAB recruitment after two patients reported sustained high-level pain (10 points in VAS pain score). Thirty-one out of 79 patients with US images suggestive of CTNs (38 nodules) had the FNAB and confirmed CTNs diagnoses (group 1). Five more patients were posteriorly excluded, after declining the call for a new cervical US evaluation for personal reasons. Therefore, 26 patients (n = 31 nodules) were enrolled for the cross-sectional US study.

Among the 206 thyroid cancer patients with lateral neck dissection, 77 subjects who had FNAB for suspicious LN on US images were selected for analysis. FNAB was performed based on nodule size: smaller axis ≥ 8 mm, or <8 mm but evidence of growth, according to the American Thyroid Association Guidelines 2015 (11). Thirty-nine out of 77 patients were excluded (18 patients with suspicious LN in the central neck compartments, and 21 patients with cytology diagnosis different from metastatic or reactive LNs), and group 2 was composed by the remaining 38 patients (n = 48 nodules). Data from pain during FNAB of group 2 subjects were not available.

**Figure 1:**
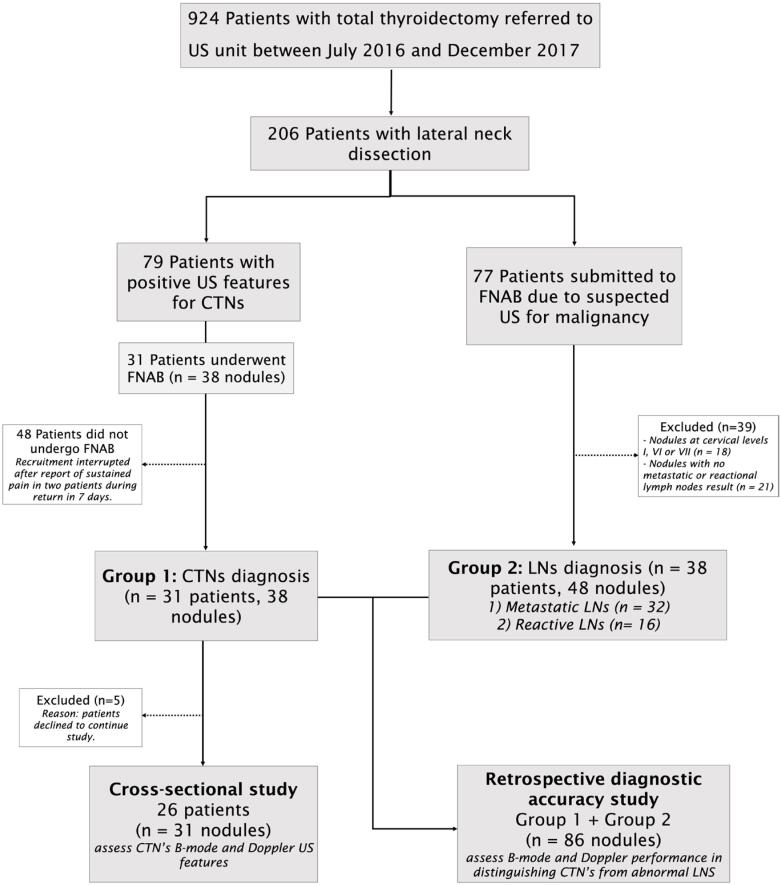
Flowchart of participant enrollment in the study. CTNs: cervical traumatic neuromas; FNAB: fine-needle aspiration biopsy; LNs: lymph nodes.

The patient selection flowchart is summarized in Figure 1.

The institutional ethics committee approved the study (nº 2.056.465). CAAE registration number: 66849717.9.0000.0065), and all participants from the cross-sectional prospective study provided written informed consent. Informed consent was waived for the use of retrospective data on diagnostic accuracy study.

### Cervical ultrasonography

The US examinations were performed using a Logic E9 machine (GE Healthcare, Waukesha, WI) equipped with a high-frequency linear transducer (9-15 MHz). Representative images of B-mode and color Doppler US from group 1 (diagnosed CTNs) and group 2 (metastatic and reactive LNs) were retrospectively selected, pooled in a digital file, and anonymized for blinded analysis. Two patients were allocated to both groups, as they had CTN and LN diagnoses in different nodules. The analysis of US images was performed by two radiologists (AAA and BBB, with 8 and 4 years of cervical US practice, respectively); any disagreements were resolved by a third radiologist. Patients with CTNs diagnosed on FNAB were prospectively scheduled for additional B-mode and color Doppler US studies by the same two independent radiologists, blinded to the patient information, including FNAB cytological results. Any disagreements were resolved by consensus.

The following B-mode and color Doppler parameters were assessed (6-8,11): location according to cervical level (14); dimensions (short, long and transverse axis), margins (regular or irregular); limits (well- or ill-defined); predominant echogenicity (hyper or iso/hypoechogenic to the sternocleidomastoid muscle); echotexture (homogeneous or heterogeneous); dystrophic calcifications; cystic component; internal echogenic foci; hypoechogenic internal lines; hyperechogenic central area; continuity with a nerve structure (hypoechogenic nonvascular cord-like structure entering the nodule); continuity to a spinal nerve (which originates from vertebral foramen); nontransected nerve passing through the nodule; and intranodular blood flow detected by color Doppler.

### Statistical analysis

The US features were analyzed with chi-squared or Fisher’s exact tests (nominal variables) and Student’s t-test for two samples (continuous variables) according to groups (CTNs *vs*. LNs) or subgroups of CTNs (diagnostic *vs.* nondiagnostic cytology). The Mann-Whitney test was used if continuous variables were not normally distributed.,

Receiver operating characteristic (ROC) curves were built to demonstrate sensitivity and specificity, and the point on the ROC curve with maximum Youden index (sensitivity – [1 – specificity]) was calculated to access the optimal cut-off point for continuous variables. The diagnostic performance to distinguish CTNs from LNs was evaluated according to sensitivity, specificity, accuracy, positive and negative likelihood ratios for each US feature, and 95% confidence intervals. Due to the low CTN prevalence, a convenience sample was used, and a statistical power level > 0.80 for the significant difference findings was obtained in the prospective study. A *P* value of less than 0.05 indicated a significant difference. Statistical analyses were performed by using R software (version 3.6.3; R Foundation for Statistical Computing, Vienna, Austria).

## RESULTS

### Patient characteristics

Sixty-seven patients (52 [41-64] years, 52 women) were included in the present study. Two patients were allocated to both groups, as they had CTN and LN diagnoses in different nodules. The mean follow-up period from lateral neck dissection to cervical nodule cytologic diagnosis was 51 (32-83) months. Patients in both groups had only thyroid neoplasms, without other concurrent malignancies. Papillary thyroid carcinoma was the most common histological type (85.1%). Among the patients with CTNs, 74.2% (23/31) had nodule-related pain before puncture. The baseline participant characteristics (Table 1) were not significantly different between groups.

**Table 1:** Baseline participant characteristics according to CTNs and LNs groups

Parameter		Total	Group 1 (CTNs)	Group 2 (LNs)	P value
Patients^a^		67	31	38	
Lateral neck nodules		86	38 (44%)	48 (56%)	
Age [median (IQR)]		52 (41-64)	50 (41-58)	55 (43-69)	.108
Sex: Woman		52 (77.6%)	25 (80.6%)	28 (73.7%)	.693
Follow-up [median (IQR)] months^b^		51 (32-83)	60 (33-107)	49 (33-73)	.252
Thyroid carcinoma					1.00
	Papillary carcinoma	57 (85.1%)	26 (83.9%)	32 (84.2%)	
	Medullary carcinoma	10 (14.9%)	5 (16.1%)	6 (15.8%)	

CTNs: cervical traumatic neuromas; LNs: lymph nodes; IQR: interquartile range.

a Two patients were allocated into both groups because they had CTNs and LNs diagnosis.

b Follow-up period from lateral neck dissection and CTN or LN diagnosis.

**Figure 2:**
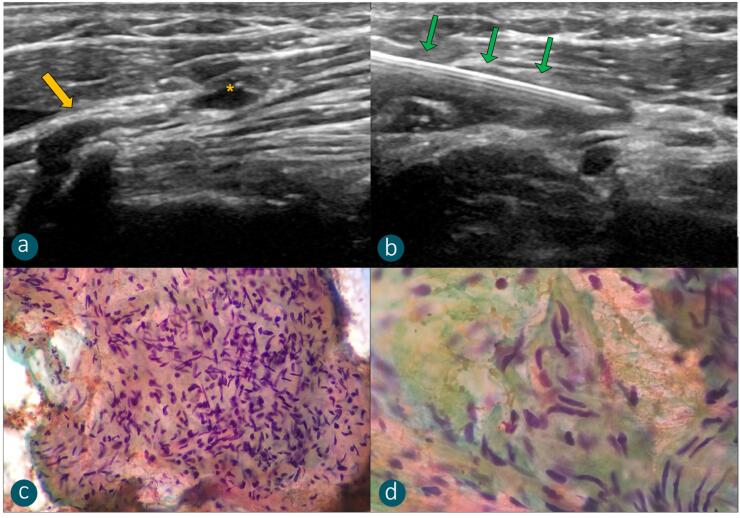
(**a-b**) 52-year-old patient after lateral neck dissection due to lymph node metastasis of papillary thyroid carcinoma presenting a cervical nodule (yellow asterisk) in continuity with the C4 spinal root (yellow arrow). FNAB was performed (green arrows), with cytopathology confirmation of CTN. Smear slide with Papanicolaou staining, magnifications of 400x (**c**) and 1000x (**d**), showing multiple cells with elongated, bluish-colored nuclei, compatible with spindle cell proliferation, a neuroma diagnostic finding.

A total of 86 nodules (38 CTNs + 48 LNs) were evaluated. Among the 38 CTNs submitted to FNAB (group 1), the presence of neural spindle cells in cytology was observed in 15 nodules (Figure 2). All patients presented pain exacerbation during puncture and undetectable thyroglobulin or calcitonin in FNAB washout, thus meeting the reference standard criteria. Nodules from group 2 were diagnosed in FNAB as metastatic LNs (n = 32) or reactive LNs (n = 16).

### Performance of US in the diagnosis of CTNs

Table 2 summarizes all US features from group 1 and group 2 nodules and the performance of each US feature in the diagnosis of CTNs is presented in Table 3.

**Table 2:** Differences in ultrasound features of lateral neck nodules diagnosed as CTNs and LNs (metastatic or reactive)

Features		CTNs (n = 38)	LNs (n = 48)	P value
Continuity with nerve structure (%)		37 (97.4)	0 (0)	< .001
Echotexture heterogeneous (%)		38 (100)	29 (60.4)	< .001
Predominant echogenicity (%)				
	Hyperechogenic	14 (36.8)	5 (10.4)	.003
	Iso or hypoechogenic	24 (63.2)	43 (89.6)	.003
Dystrophic calcification (%)		0 (0)	1 (2.1)	1.00
Internal echogenic dots (%)		28 (73.7)	25 (52.1)	.001
Hypoechogenic internal lines (%)		34 (89.5)	1 (2.1)	<.001
Central hyperechoic area (%)		8 (21.1)	1 (2.1)	.012
Echogenic hilar line absent (%)		38 (100)	44 (91.7)	.191
Morphology (%)				
	Oval or round	4 (10.5)	43 (89.6)	<.001
	Elongated or fusiform	34 (89.5)	5 (10.4)	<.001
Defined limits (%)		24 (63.2)	41 (85.4)	.017
Regular margins (%)		26 (68.4)	27 (56.3)	.249
Absence of cystic component (%)		38 (100)	43 (89.6)	.113
Doppler vascularization pattern (%)				
	Usual hilar	0 (0)	16 (33.3)	<.001
	Central and/or peripheral	0 (0)	24 (50)	<.001
	Absent	36 (94.7)	8 (16.7)	<.001
Short axis (cm)^a^		0.41 (0.36-0.48)	0.72 (0.52 - 1.01)	<.001
Long axis (cm)^a^		1.36 (1.13-1.84)	1.19 (0.96 - 1.67)	.137
Short/Long axis ratio^a^		0.30 (0.23-0.37)	0.61 (0.49 - 0.71)	<.001

CTNs: cervical traumatic neuromas; LNs: lymph nodes.

a Data are median and in parentheses the 25th and 75th percentiles.

**Table 3:** Performance of ultrasound features to diagnose CTNs

Features	Nodules N°		Sensitivity (%)	Specificity (%)	Accuracy (%)	Positive Likelihood Ratio	Negative Likelihood Ratio
	CTNCs	LNs					
Continuity with nerve structure	37	0	97.4 (86.2-99.9)	100 (92.6-100)	98.8 (93.7-100)	*	0.0 (0.0-0.2)
Hypoechogenic internal lines	34	1	89.5 (75.2-97.1)	97.9 (88.9-100)	94.2 (86.9-98.1)	42.9 (6.2-299.6)	0.1 (0.0-0.3)
Short/Long axis ratio ≤ 0,42	33	2	86.8 (71.9-95.6)	95.8 [85.7-99.5]	91.9 (83.9-96.7)	20.8 (5.3-81.4)	0.1 (0.1-0.3)
Fusiform morphology	34	5	89.5 (75.2-97.1)	89.6 (77.3-96.5)	89.5 (81.1-95.1)	8.6 (3.7-19.8)	0.1 (0.0-0.3)
Absent Doppler vascularization	36	8	94.7 (82.2-99.4)	83.3 (69.8-92.5)	88.4 (79.6-94.3)	5.7 (3.0-10.7)	0.1 (0.0-0.2)
Short axis ≤ 0,48 cm	29	3	76.3 (59.8-88.6)	93.7 (82.8-98.7)	86.1 (76.9-92.6)	12.2 (4.0-37.0)	0.2 (0.1-0.4)
Heterogeneous echotexture	38	29	100 (90.7-100)	39.6 (25.8-54.7)	66.3 (55.3-76.1)	1.7 (1.3-2.1)	0.00
Hyperechogenic	14	5	36.8 (21.8-54.0)	89.6 (77.3-96.5)	66.3 (55.3-76.1)	3.5 (1.4-8.9)	0.7 (0.5-0.9)
Central hyperechoic area	8	1	21.1 (9.6-37.3)	97.9 (88.9-100)	63.9 (52.9-74.0)	10.1 (1.3-77.3)	0.8 (0.7-1.0)
Internal echogenic dots	28	25	73.7 (56.9-86.6)	47.9 (33.3-62.8)	58.3 (48.2-69.8)	1.4 (1.0-2.0)	0.5 (0.3-1.0)

Note. Numbers in parentheses are the 95% confidence interval. The sonographic characteristics were ordered according to the highest accuracy.

* Value not calculated due to zero denominator.

CTNs: Cervical traumatic neuromas; LNs: lymph node diseases.

Direct continuity with a nerve structure showed the best performance in distinguishing CTNs from LNs, with 97% sensitivity and 100% specificity. This feature was observed in 37 (97.4%) CTNs but no LNs (P < 0.001). Hypoechogenic internal lines presented 89% sensitivity and 98% specificity in CTN diagnosis and were detected in 34 (89.5%) CTNs and one (2.1%) LN (P < 0.001).

**Figure 3:**
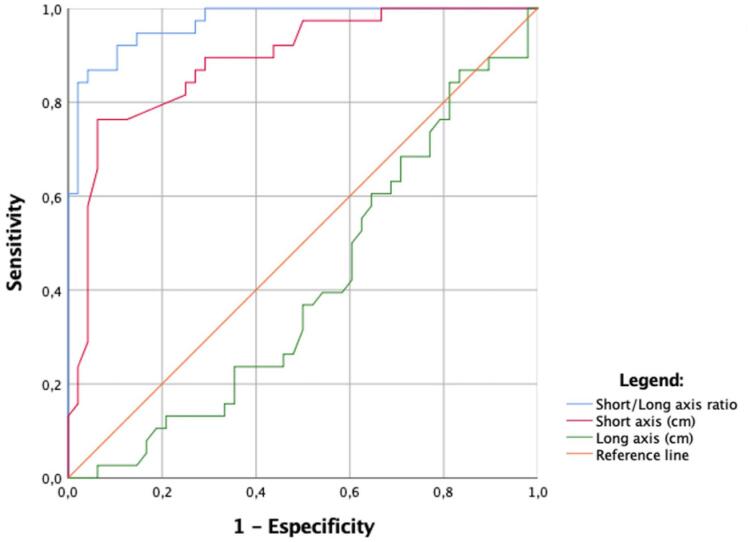
Receiver operating characteristic (ROC) curves show the short axis, long axis and short/long axis ratio dispersion slopes for CTN detection. Areas under the ROC curve were 0.97 (95% CI: 0.94 - 1.0; P<0.01) for the short/long axis ratio, 0.89 (95% CI: 0.82 - 0.96; P<0.01) for the short axis, and 0.41 (95% CI: 0.29 – 0.53; P=0.14) for the long axis.

The median short axis and the median short/long axis ratio were significantly smaller in CTNs than in LNs (0.41 cm *vs.* 0.72 cm, P = < 0.001; 0.30 *vs.* 0.61, P = < 0.001). The ROC curve and areas under the curve of sensitivity and 1-specificity from short/long axis ratio, short axis and long axis variables are shown in Figure 3. The best ROC curve cutoff values for the short axis and the short/long axis ratio to distinguish CTNs from LNs were 0.48 cm and 0.42, respectively, both presenting accuracy above 80% to predict neuromas.

Most CTNs (94.7%) did not present color Doppler vascularization, while only 8 LNs (16,7%) had absent nodule vascularization. A small vessel entering the nodule was detected in two neuromas. Absent Doppler vascularization presented 83% sensitivity and 88% specificity in CTN diagnosis.

### CTNs ultrasound features

Thirty-one CTNs nodules were included in the prospective cross-sectional ultrasound study, 11 of whom (35.5%) had diagnostic cytology due to the presence of neural spindle cells, and the remaining 20 CTNs (64.5%) had inconclusive cytology due to scarce or hemorrhagic cellular material. Neuromas were dichotomized into subgroups based on the cytological results. A comparison of the diagnostic and inconclusive cytology subgroups showed a significant difference in echogenicity (P = 0.008): all diagnostic cytology nodules (11/11; 100%) were iso/hypoechogenic; on the other hand, among CTNs with inconclusive cytology, 55% (11/20) were hyperechogenic. The nodule dimensions were significantly higher in the inconclusive cytology CTNs: mean short axis (0.5 cm *vs.* 0.39 cm; P = 0.031); mean transverse axis (1.05 cm *vs.* 0.74 cm; P = 0.005); and mean long axis (2.35 cm *vs.* 1.64 cm; P = 0.021).

All 31 CTNs evaluated in the prospective cross-sectional ultrasound showed evidence of continuity with a nervous structure. This continuity was from a spinal root in 29 neuromas (93.5%), mostly C3 (51.6%) and C4 (58.1%). Eight CTNs (26%) had continuity with two or more spinal roots. The majority of CTNs (n = 28; 90.3%) had a noninterrupted nerve passing through the nodule. All 31 CTNs had heterogeneous echotexture and echogenic hilar line absent, and none presented cystic components.

## DISCUSSION

Cervical traumatic neuromas are infrequent but relevant findings in US exams of thyroid carcinoma patients submitted to lateral neck dissection. The present study revealed that US is an excellent diagnostic method for evaluating cervical traumatic neuromas and the assertive radiologic diagnosis avoids painful FNABs.

The prevalence of CTNs after lateral neck dissection is probably underestimated, reported in 1.1 to 17.8% (4-7). The variable frequency could be attributed to different diagnostic methods and the complexity related to cervical US examination of postoperative conditions, especially the difficulty in detecting the continuity of the cervical nodule with neural structure. The CTN prevalence in the present study was 38.3% (79 suggestive CTNs patients/206 patients with lateral neck dissection), a high rate probably justified by trained US examiners in cervical neural structure recognition, combined with high-resolution ultrasound machines.

Continuity of the cervical nodule with a nerve was the main finding for CTN recognition, corroborating previous studies (6,15). Although continuity with the nerve is a crucial anatomical feature of neuromas, it is often very discreet on US and the examiner may neglect it, if not trained to detect it. The identification of hypoechogenic internal lines in CTNs also contributes significantly to their differential diagnosis from LNs. Similar to hypoechogenic tubular structures in US studies of peripheral nerves (16-18), they probably correspond to the CTNs nerve fascicles (6).

**Figure 4:**
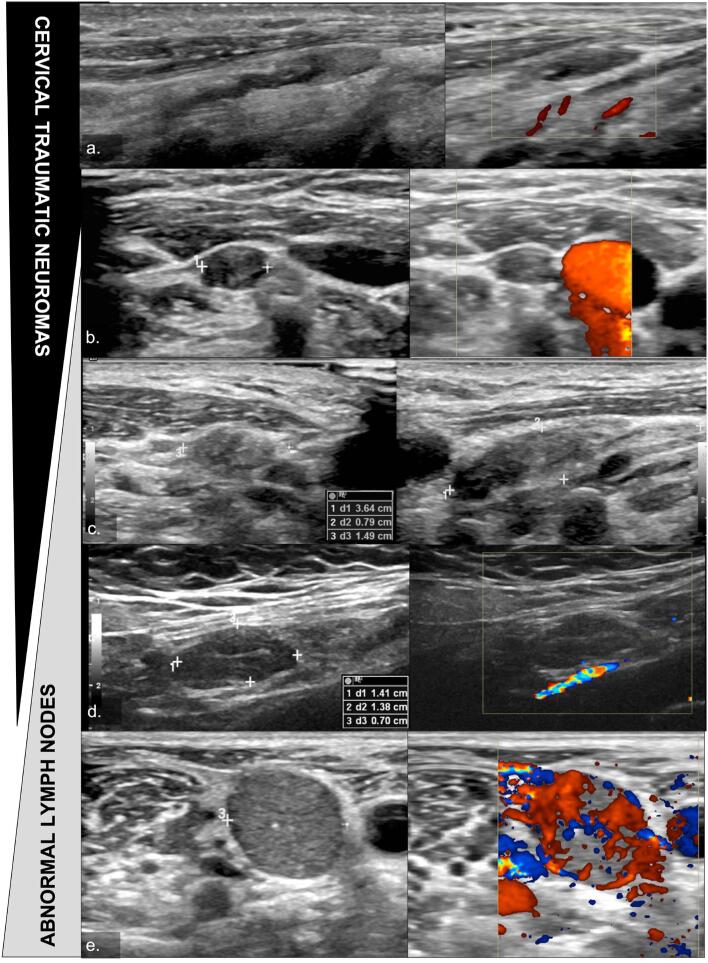
CTNs US features and their distinction from abnormal LNs. (**a**) Typical CTN in continuity with the cervical nerve, fusiform morphology, and no Doppler vascularization. (**b**, **c**) CNTs that can be confused with metastatic LNs on US, as they present an absence of evident communication with neural structure in this image, oval morphology in the transverse axis, and prominent short axis (0.79 cm in image c). (**d**) Metastatic LN with similar features to the CTNs shown in c and d, presenting fusiform morphology, no communication with neural structure and no Doppler vascularization. (**e**) Typical metastatic LN, with rounded morphology, microcalcification, and exuberant peripheral and central vascularization on color Doppler study.

Metastatic lymph nodes are usually round-shapes and a short axis > 0.8 cm increases the risk of malignancy in a cervical lateral neck nodule (11,19). Herein, nodule dimensions contributed to the differentiation oof CTNs from LNs, especially short/long ratios. The CTNs were mainly elongated nodules with a mean short axis < 0.5 cm. Despite the heterogeneous echotexture and the absence of echogenic hilar line, which may be misdiagnosed as suspicious LNs by an inexperienced examiner, the fusiform shape indicates the presence of a CTN (Figure 4). In addition, most neuromas did not present evidence of Doppler vascularization, as also reported by Ha and cols. (2012). However, the detailed color Doppler evaluation performed in the cross-sectional study evidenced small vessels inside two CTNs, a vascularization pattern different from the subcapsular or central vascularization often observed in metastatic LNs (19).

**Figure 5:**
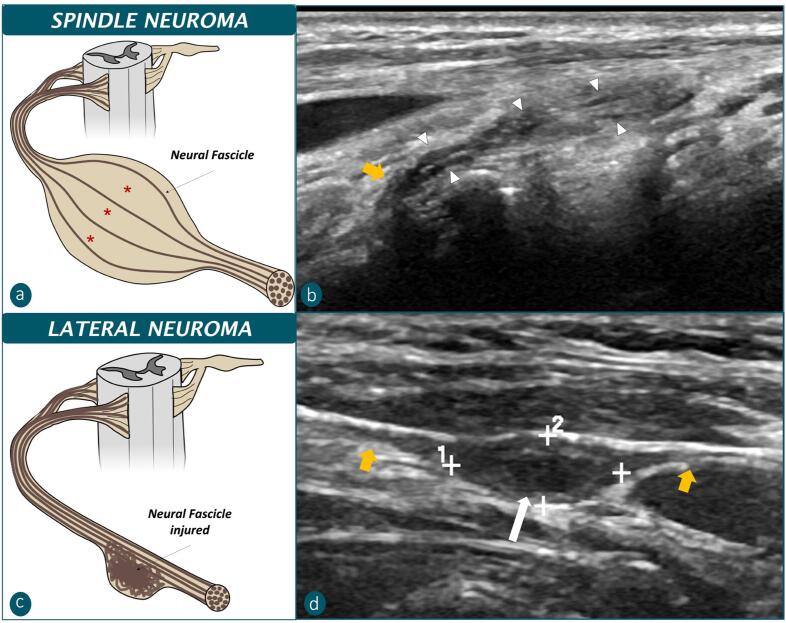
Correlation between Sunderland’s types of neuromas in nontransected nerves and CTN echogenicity patterns. **a**) Spindle neuroma, bulbar enlargement determined by an increase in interfascicular connective tissue (red asterisks). **b**) Hyperechogenic CTN, which has hypoechogenic internal lines/neural fascicles (arrowheads) more separated from each other by echogenic tissue in bulbar enlargement than in the spinal nerve (yellow arrow), similar to spindle neuromas. **c**) Lateral neuroma: some injured fascicles allow neural expansion in the interfascicular tissue. **d**) Hypoechogenic CTN, with bulbar enlargement of the cervical nerve (yellow arrows), which has little intermingled hyperechogenic tissue, and an enlarged hypoechogenic internal line (white arrow), similar to lateral neuromas.

Cytology evaluation of CTNs is frequently non-diagnostic. In the present study, 60% of performed CTNs FNABs were inconclusive. In US examination, CTNs with inconclusive cytology were significantly larger and predominantly hyperechogenic compared to those in which neural spindle cells were recognized in cytology. Sunderland proposed two types of neuromas in non-sectioned nerves (Sunderland, 1968): 1) the spindle type, in which the perineurium is preserved and the connective tissue around the fascicles is the main component responsible for its nodular increase; and 2) the lateral type, in which the perineurium of some fascicles is injured and the regenerating axon grows into interfascicular tissue after escaping through the ruptured fascicle. In US, nerve fascicles are reported as hypoechogenic tubular structures surrounded by echogenic connective perifascicular tissue. Therefore, it could be inferred that spindle-type neuromas appear predominantly as hyperechogenic nodules due to abundant interfascicular tissue. As the neural tissue is protected by epineurium inside the fascicles, FNABs of spindle-type neuromas would be more frequently non-diagnostic. On the contrary, the lateral-type neuromas could correspond to hypoechogenic nodules due to the neural axons in the interfascicular tissue unprotected by the perineurium. The ruptured fascicle would contribute to successful aspiration of neural cells in FNABs of lateral-type neuromas (Figure 5).

In the present study, the US features with the best sensitivity and specificity (>80%) to distinguish CTNs from abnormal LNs were the continuity of the nodule with a nervous structure, the presence of hypoechogenic internal lines, a fusiform morphology with short/long axis ratio ≤ 0.42 and the absence of vascularization at Doppler evaluation. The combination of these US features all together were not observed in any lymph nodes, and therefore, could be considered a CTN signature in US, avoiding painful FNAB to confirm the diagnosis.

The present study represents a cross sectional evaluation of a cohort of thyroid carcinoma patients followed in the same reference center. However, due to the low prevalence of CTNs related to lateral neck dissection, a small number of patients were included. The retrospective design of the diagnostic accuracy study, with different inclusion criteria for CTN- or LN-diagnosed patients, especially continuity with a neural structure and the nodule’s short axis used as eligibility criteria for the CTN and LN groups, respectively, may overestimate the sensitivity and specificity of accuracy tests. The absence of postoperative CTN pathological analysis also limits the strength of imaging and pathology correlations.

US is a very useful method for assessing CTNs, presenting high performance in distinguishing them from abnormal LNs in patients after lateral neck dissection. Continuity with the nervous structure and hypoechogenic internal lines presented the highest overall accuracy to predict CTNs. Radiologists can play a central role in identifying CTNs when performing high-quality cervical US in postoperative patients with metastatic thyroid cancer, avoiding unnecessary and painful invasive procedures.
